# Sex modifies the predictive value of computed tomography combined with serum potassium for primary aldosteronism subtype diagnosis

**DOI:** 10.3389/fendo.2023.1266961

**Published:** 2023-11-16

**Authors:** Yingxing Wu, Zuxiang Wu, Jingan Rao, Huan Hu, Zhiqiang Chen, Chenkai Hu, Qiang Peng, Ping Li

**Affiliations:** Department of Cardiovascular Medicine, The Second Affiliated Hospital of Nanchang University, Nanchang, Jiangxi, China

**Keywords:** primary aldosteronism, subtype diagnosis, computed tomography, serum potassium, sex differences

## Abstract

**Objective:**

We aimed to investigate the predictive value of the CT findings combined with serum potassium levels for primary aldosteronism (PA) subtype diagnosis, with a particular interest in sex differences.

**Methods:**

In this retrospective study, we eventually included 482 PA patients who underwent successful adrenal venous sampling (AVS) and had available data. We diagnosed the subjects as having either unilateral (n = 289) or bilateral PA (n = 193) based on AVS. We analyzed the concordance rate between AVS and adrenal CT combined with serum potassium and performed a logistic regression analysis to assess the prevalence of unilateral PA on AVS.

**Results:**

The total diagnostic concordance rate between CT findings and AVS was 51.5% (248/482). The prevalence of hypokalemia in men and women was 47.96% (129/269) and 40.85% (87/213), respectively. The occurrence of unilateral lesions on CT and hypokalemia was significantly associated with an increased prevalence of unilateral PA [odds ratio (OR) 1.537; 95% confidence interval (CI) 1.364–1.731; *p* < 0.001]. In male participants, G2 (bilateral lesion on CT and normokalemia), G3 (unilateral lesion on CT and normokalemia), G4 (bilateral normal on CT and hypokalemia), G5 (bilateral lesion on CT and hypokalemia), and G6 (unilateral lesion on CT and hypokalemia) were significantly increased for the prevalence of unilateral PA on AVS (G2: OR 4.620, 95% CI 1.408–15.153; G3: OR 6.275, 95% CI 2.490–15.814; G4: OR 3.793, 95% CI 1.191–12.082; G5: OR 16.476, 95% CI 4.531–59.905; G6: OR 20.101, 95% CI 7.481–54.009; all *p* < 0.05), compared with G1 (patients with bilateral normal on CT and normokalemia). However, among female participants, we found an increased likelihood for unilateral PA in patients with unilateral lesions on CT and hypokalemia alone (OR 10.266, 95% CI 3.602–29.259, *p* < 0.001), while no associations were found in other groups (all *p* > 0.05). Sex had a significant effect on modifying the relationship between unilateral PA and the combination of CT findings and serum potassium (*p* for interaction <0.001).

**Conclusion:**

In conclusion, our results indicated that CT findings combined with serum potassium levels have a great value for predicting the subtype of PA and are stronger in men.

## Introduction

Primary aldosteronism (PA) is a frequent cause of secondary hypertension due to autonomous aldosterone secretion by the adrenal glands. The prevalence of PA in hypertensive patients is reportedly 3.2%–12.7% in primary care centers and 1%–29.8% in referral centers (1). Patients with PA have a higher prevalence of various target organ damage and cardiovascular complications such as stroke, microalbuminuria, left ventricular hypertrophy, myocardial infarction, atrial fibrillation, and heart failure than those with essential hypertension (2–4). Aldosterone-producing adenoma (APA) and idiopathic hyperaldosteronism (IHA) are the most frequent forms of PA, accounting for 30% and 60% of all cases, respectively (5). A differential diagnosis between these subtypes is a crucial step in diagnosis and choice of treatment, as most patients with APA are surgically curable, while patients with IHA require lifelong treatment with mineralocorticoid receptor (MR) antagonists (6).

Although adrenal venous sampling (AVS) is recommended as the gold standard method for classifying subtypes by the current guidelines (6, 7); this approach is invasive, costly, technically challenging, and limited to specialized centers (8, 9). Therefore, a simpler non-invasive method for subtype classification is needed. Adrenal computed tomography (CT) is a widely used imaging technique in most centers and is more convenient and inexpensive than AVS. The current guidelines recommend that all patients with PA undergo adrenal CT to exclude adrenocortical carcinoma and severing as the initial study for subtype testing. Nevertheless, previous studies have shown that the diagnostic accuracy of adrenal CT for subtype classifying is not sufficient compared with AVS (10–12). Based on clinical variables, several prediction models have been reported to distinguish among differing subtypes for use instead of AVS (13–19). Although the studies incorporated different clinical parameters, CT findings and serum potassium served as shared predictors for subtype diagnosis of PA patients. A recent study proposed graded recommendation strength of AVS indication based on the probability of unilateral hyperaldosteronism on AVS determined by CT findings and serum potassium (12). However, it is not clear whether there was a significant difference in the association between the subtype of PA and the combination of CT findings and serum potassium between male and female populations.

In this study, we aimed to investigate the predictive value of the CT findings combined with serum potassium levels for subtype diagnosis, with a particular interest in sex differences.

## Methods

### Study participants

Our study included 482 PA patients with available data on clinical characteristics, biochemistry, CT findings, and successful AVS from November 2017 to October 2021 at the Second Affiliated Hospital of Nanchang University. The diagnostic procedures for PA followed the Endocrine Society Clinical Practice Guidelines (6) and the Expert Consensus on the Diagnosis and Treatment of Primary Aldosteronism in China (7) and were described in detail in our previous study (20). The Medical Research Ethics Committee of the Second Affiliated Hospital of Nanchang University reviewed and approved the study and waived the need for informed consent.

The inclusion criteria of this study were as follows: a) patient aged between 18 and 75 years, b) confirmed diagnosis of PA, c) AVS was bilaterally successful, and d) availability of data on clinical characteristics, biochemistry, and CT findings. Patients with other forms of secondary hypertension, such as renal artery stenosis, hyperthyrea, Cushing’s syndrome, or pheochromocytoma, were excluded.

### Diagnosis criteria for PA

Case detection of PA was performed using plasma aldosterone concentration (PAC) to plasma renin activity (PRA) ratio (ARR). Before the screening test, diuretics and mineralocorticoid receptor antagonists were withdrawn for at least 4 weeks, and dihydropyridine calcium channel blockers, angiotensin-converting enzyme inhibitors, angiotensin II receptor blockers, and β-adrenergic receptor blockers were discontinued for at least 2 weeks. Antihypertensive medications were replaced by non-dihydropyridine calcium channel blockers (verapamil slow-release) and/or α-adrenergic receptor blockers (terazosin hydrochloride) for uncontrolled hypertension, and patients were required to monitor blood pressure periodically. Blood samples for PRA and PAC were collected at 7 a.m. after the patients had been upright for 2 hours. The screening test was considered positive when the ARR > 30 (ng/dl)/(ng/ml/h) coupled with PAC >15 ng/dl. For patients with positive screening tests, the captopril challenge test (CCT) or saline infusion test (SIT) was performed to further confirm the diagnosis of PA. When the aldosterone suppression rate was less than 30% or PAC was greater than 11 ng/dl after 2 hours of oral administration of 50 mg captopril, CCT was regarded as positive. The cutoff value for SIT was PAC > 10 ng/dl after infusion of 2L 0.9% saline over 4 hours in the seated position (6, 7).

### Serum potassium levels

Hypokalemia was defined as a serum potassium level of <3.5 mmol/L in the available potassium data during hospitalization.

### CT imaging

All the patients were examined with thin-slice adrenal CT (GE Co., Chicago, IL, USA). In the absence of AVS outcomes, the adrenal CT images were individually evaluated by two experienced radiologists. A panel discussion serves to settle disagreements between the two radiologists. The CT findings were classified into bilateral normal, unilateral lesion, and bilateral lesion. The adrenal gland’s normal appearance (the thickness of the adrenal gland was <10 mm) on both sides was described as the bilateral normal of CT findings. Unilateral adrenal lesion of CT findings was defined as unilateral adenoma, nodule, or hyperplasia without abnormal appearance on the contralateral glands.

### AVS procedure and subtype diagnosis

The bilaterally simultaneous catheterization AVS without adrenocorticotropic hormone (ACTH) stimulation was performed in all patients according to the current guidelines (8). The right adrenal vein (RAV) and left adrenal vein (LAV) were catheterized through the percutaneous right forearm vein approach and femoral vein approach under fluoroscopic guidance, respectively, and successful catheterization was confirmed by slow injection of a small amount of contrast medium. Blood samples were simultaneously collected from the bilateral adrenal veins, and then inferior vena cava (IVC) samples were taken as a control. Adrenal vein cannulation was regarded as successful if the selectivity index (SI) was >2, as the ratio of cortisol concentration of the adrenal vein to the IVC. The lateralization index (LI) was defined as the ratio of aldosterone to cortisol ratio on the dominant side to the non-dominant side. The criterion used for unilateral hyperaldosteronism was LI greater than 2.

### Assay methods

Assay methods were described in our previous study (20). PAC (ng/dl) and PRA (ng/ml/h) were determined by chemiluminescence immunoassay (CLIA) following the manufacturer’s instruction (Maglumi 4000 plus, Sinbe Co., Ltd., Shenzhen, China). Plasma cortisol concentration (PCC) (μg/dl) was measured by CLIA following the manufacturer’s instruction (ADVIA Centaur XP, Siemens Co., Munich, Germany).

### Statistical analysis

IBM SPSS Statistic version 23.0 (SPSS Inc., Chicago, IL, USA) was performed in the whole statistical analysis. Normally distributed continuous variables are reported as means ± standard deviations (SDs) and analyzed by unpaired *t*-test. Continuous variables with a skewed distribution are expressed as medians with interquartile range (IQR) and analyzed by the Mann–Whitney *U*-tests. Categorical variables are presented as absolute numbers with percentages and analyzed by *χ*^2^ test or Fisher’s exact tests. The population characteristics were compared among patients with unilateral and bilateral PA by sex classification. CT findings (unilateral lesion, bilateral lesion, and bilateral normal on CT) and serum potassium (hypokalemia and normokalemia) were investigated in the subtype diagnosis of PA determined by AVS. Multivariate logistic regression analysis was used to assess the odds ratios (ORs) and 95% confidence interval (CI) of the association between unilateral PA and the combination of CT findings and serum potassium in different sexes. Patients with bilateral normal CT and normokalemia were defined as the reference group. Three models were constructed for regression analysis: Model 1 was adjusted for none; Model 2 was adjusted for age, sex (only for the overall population), body mass index (BMI; kg/m^2^), diabetes, estimated glomerular filtration rate (eGFR; ml/min/1.73 m^2^), systolic blood pressure (SBP; mmHg), and diastolic blood pressure (DBP; mmHg). Model 3 was adjusted for all covariables in Model 2 and adjusted for total cholesterol (TC; mmol/L), triglyceride (TG; mmol/L), high-density lipoprotein (HDL; mmol/L), low-density lipoprotein (LDL; mmol/L), fasting plasma glucose (FPG; mmol/L), glycosylated hemoglobin (HbA1c; mmol/L), and homocysteine (Hcy, mmol/L). Furthermore, the interactions of the association between unilateral PA and the combination of CT findings and serum potassium with age (<60 *vs.* ≥60 years), BMI (<24 *vs.* ≥24 kg/m^2^), eGFR (<90 *vs.* ≥90 ml/min/1.73 m^2^), and diabetes (yes *vs.* no) in different sex were evaluated by interaction test. *p* < 0.05 (two-tailed) was defined as a statistically significant difference.

## Results

### Baseline characteristics of study participants

A total of 482 PA patients with successful AVS were included in this study (average age 49.8 ± 10.2 years; 55.81% males). Among the participants, 289 (59.96%) were unilateral PA patients. The average age and the prevalence of hypokalemia in 269 male participants were 49.3 ± 10.2 years and 47.96% (129/269), respectively. The average age and the prevalence of hypokalemia in 213 female participants were 50.5 ± 10.1 years and 40.85% (87/213), respectively. The prevalence of unilateral PA in male and female participants was 60.59% (163/269) and 59.15% (126/213), respectively.

The distributions of study population baseline characteristics according to the subtype of PA among male and female participants are presented in **Table 1**. In the male group, the unilateral PA patients had higher values for SBP, serum sodium, PAC, and ARR and lower values for TC, LDL, serum potassium, and PRA, compared to bilateral PA patients. Female participants with unilateral PA had lower serum potassium levels compared with bilateral PA. However, no significant differences were found in SBP, TC, LDL, PRA, PAC, ARR, and serum sodium in female participants among unilateral and bilateral PA patients.

**Table 1:** Comparison of baseline characteristics in patients.

Variable	Total (n = 482)	Male		*p*-Value^a^	Female		*p*-Value^b^
		Unilateral (n = 163)	Bilateral (n = 106)		Unilateral (n = 126)	Bilateral (n = 87)	
Age (years)	49.8 ± 10.2	49.6 ± 10.5	48.8 ± 9.8	0.507	50.2 ± 10.3	51.0 ± 9.8	0.545
BMI (kg/m^2^)	25.87 ± 3.58	26.69 ± 3.15	27.02 ± 3.55	0.425	24.69 ± 3.81	24.63 ± 3.15	0.915
Diabetes [n (%)]	55 (11.4)	23 (14.1)	13 (12.3)	0.650	11 (8.7)	8 (9.2)	0.907
SBP (mmHg)	156.52 ± 20.64	158.61 ± 19.30	153.39 ± 21.19	0.038	157.88 ± 21.16	154.42 ± 21.32	0.244
DBP (mmHg)	94.66 ± 14.73	96.46 ± 14.51	94.77 ± 14.27	0.349	93.68 ± 15.29	92.56 ± 14.72	0.594
TC (mmol/L)	4.66 ± 1.06	4.35 ± 0.92	4.69 ± 0.99	0.004	4.97 ± 1.13	4.76 ± 1.16	0.203
TG (mmol/L)	1.99 ± 1.64	2.16 ± 1.63	2.30 ± 2.19	0.544	1.81 ± 1.39	1.55 ± 0.96	0.134
HDL (mmol/L)	1.13 ± 0.33	1.00 ± 0.23	1.00 ± 0.27	0.974	1.30 ± 0.39	1.28 ± 0.31	0.650
LDL (mmol/L)	2.68 ± 0.87	2.48 ± 0.74	2.78 ± 0.82	0.002	2.86 ± 0.92	2.67 ± 1.01	0.152
Hcy (mmol/L)	13.33 ± 5.33	15.38 ± 5.80	14.62 ± 5.91	0.308	10.96 ± 3.62	11.16 ± 3.08	0.686
FPG (mmol/L)	5.44 ± 1.71	5.59 ± 1.70	5.45 ± 2.19	0.555	5.37 ± 1.61	5.24 ± 1.12	0.513
HbA1c (%)	5.58 ± 0.80	5.57 ± 0.81	5.64 ± 0.90	0.551	5.58 ± 0.82	5.52 ± 0.60	0.587
eGFR (ml/min/1.73 m^2^)	93.85 ± 25.79	88.39 ± 25.46	88.62 ± 19.59	0.933	101.96 ± 29.12	98.68 ± 24.27	0.388
Cr (μmol/L)	78.69 ± 26.95	92.00 ± 27.93	89.39 ± 19.44	0.368	62.80 ± 21.06	63.74 ± 20.37	0.745
UA (μmol/L)	365.30 ± 94.73	406.39 ± 90.80	408.81 ± 85.94	0.827	312.32 ± 73.68	312.02 ± 72.33	0.977
Serum potassium (mmol/L)	3.53 ± 0.51	3.33 ± 0.51	3.72 ± 0.41	<0.001	3.49 ± 0.52	3.73 ± 0.43	<0.001
Serum sodium (mmol/L)	141.31 ± 2.32	142.05 ± 2.22	141.41 ± 2.25	0.024	140.74 ± 2.36	140.64 ± 2.13	0.841
PRA (ng/ml/h)	0.16 (0.02, 0.46)	0.13 (0.02, 0.42)	0.19 (0.06, 0.50)	0.023	0.12 (0.02, 0.45)	0.22 (0.03, 0.50)	0.103
PAC (ng/dl)	26.14 (20.94, 35.74)	29.08 (21.82, 42.82)	22.90 (18.89, 28.61)	<0.001	26.98 (21.70, 34.81)	27.24 (21.42, 34.35)	0.540
ARR (ng/dl)/(ng/ml/h)	162.00 (57.18, 1,098.38)	273.33 (63.13, 1,707.50)	119.23 (50.84, 421.35)	0.001	301.73 (67.86, 1,181.93)	99.76 (53.23, 857.00)	0.055

BMI, body mass index; SBP, systolic blood pressure; DBP, diastolic blood pressure; TC, serum total cholesterol; TG, triglyceride; HDL, high-density lipoprotein; LDL, low-density lipoprotein; Hcy, homocysteine; FPG, fasting plasma glucose; HbA1c, glycosylated hemoglobin; eGFR, estimated glomerular filtration rate; Cr, creatinine; UA, uric acid; PRA, plasma renin activity; PAC, plasma aldosterone concentration; ARR, aldosterone to renin ratio.

a Comparison between unilateral and bilateral PA in male participants.

b Comparison between unilateral and bilateral PA in female participants.

### Diagnostic concordance rate between CT findings and AVS

The proportion of bilateral normal, bilateral lesion, and unilateral lesion on adrenal CT findings was 31.1% (150/482), 15.1% (73/482), and 53.7% (259/482), respectively. The diagnostic concordance rate between AVS and bilateral normal, bilateral lesion, and unilateral lesion on adrenal CT findings was 62.0% (93/150), 37.0% (27/73), and 49.4% (128/259), respectively. Although adrenal CT was recommended as the initial study for subtype testing, the overall diagnostic concordance rate of CT findings was 51.5% (248/482). Moreover, approximately 38.0% (57/150) of patients with bilateral normal CT showed unilateral PA on AVS. In addition, 22.4% (58/259) of patients with unilateral lesion on CT performed exhibited hyperaldosteronism in the contralateral adrenal gland on AVS (**Table 2**).

**Table 2:** Diagnostic concordance rate between CT finding and AVS.

CT finding	Outcomes of AVS			Concordance of CT finding, % (n/N)	Prevalence of unilateral PA on AVS, % (n/N)
	Bilateral	Unilateral			
		Left	Right		
Bilateral (n = 223)					
Normal (n = 150)	93	14	43	62.0 (93/150)	38.0 (57/150)
Lesion (n = 73)	27	22	24	37.0 (27/73)	63.0 (46/73)
Unilateral (n = 259)					
Left (n = 196)	63	84	49	49.4 (128/259)	71.9 (186/259)
Right (n = 63)	10	9	44		
Total				51.5 (248/482)	60.0 (289/482)

AVS, adrenal venous sampling; PA, primary aldosteronism.

### Serum potassium levels in patients with different AVS outcomes

The prevalence of hypokalemia was 56.7% (164/289) in patients with unilateral PA on AVS but was 29.0% (56/193) in patients with bilateral PA on AVS. Furthermore, the prevalence of unilateral PA on AVS was 74.5% in patients with hypokalemia. Serum potassium levels were correlated with the subtype diagnosis of PA (*p* < 0.001) (**Table 3**).

**Table 3:** Serum potassium levels in patients with different AVS outcomes.

	Outcomes of AVS		Prevalence of unilateral PA on AVS,% (n/N)
	Bilateral	Unilateral	
Potassium status			
Normokalemia (n = 262)	137	125	47.7 (125/262)
Hypokalemia (n = 220)	56	164	74.5 (164/220)
Prevalence of hypokalemia, % (n/N)	29.0 (56/193)	56.7 (164/289)	

AVS, adrenal venous sampling; PA, primary aldosteronism.

### Association between unilateral hyperaldosteronism and the combination of CT findings and serum potassium

The results of the multivariate logistic regression analysis are shown in **Table 4**. Overall, there was a significant association between unilateral PA and the combination of CT findings and serum potassium (*p* < 0.001). Compared with those in G1 (patients with bilateral normal on CT and normokalemia), the adjusted odds ratios for unilateral PA on AVS in G2 (a bilateral lesion on CT and normokalemia), G3 (unilateral lesion on CT and normokalemia), G4 (bilateral normal on CT and hypokalemia), G5 (bilateral lesion on CT and hypokalemia), and G6 (unilateral lesion on CT and hypokalemia) were 2.423 (95% CI: 1.079, 5.443), 3.289 (95% CI: 1.810, 5.975), 2.646 (95% CI: 1.220, 5.742), 5.767 (95% CI: 2.224, 14.952), and 10.258 (95% CI: 5.355, 19.653), respectively (all *p* < 0.05). In the stratified analysis, we found that the association of unilateral PA on AVS with the combination of CT findings and serum potassium was stronger in male participants, compared with female participants. In male participants, G2, G3, G4, G5, and G6 were significantly increased for the prevalence of unilateral PA on AVS (G2: OR 4.620, 95% CI 1.408–15.153; G3: OR 6.275, 95% CI 2.490–15.814; G4: OR 3.793, 95% CI 1.191–12.082; G5: OR 16.476, 95% CI 4.531–59.905; G6: OR 20.101, 95% CI 7.481–54.009; all *p* < 0.05), compared with G1. However, among female participants, we found an increased likelihood for unilateral PA in patients with unilateral lesions on CT and hypokalemia alone (OR 10.266, 95% CI 3.602–29.259, *p* < 0.001), while no associations were found in other groups (all *p* > 0.05). Sex had a significant effect on modifying the relationship between unilateral PA and the combination of CT findings and serum potassium (*p* for interaction <0.001).

**Table 4:** The odds ratio for unilateral PA on AVS by categorizing the combination of CT findings and serum potassium levels.

	Model 1 OR (95% CI), *p*-value	Model 2 OR (95% CI), *p*-value	Model 3 OR (95% CI), *p*-value
All participants			
Total	1.510 (1.356, 1.682), <0.001	1.510 (1.353, 1.687), <0.001	1.537 (1.364, 1.731), <0.001
G1	Reference	Reference	Reference
G2	2.471 (1.167, 5.231), 0.018	2.613 (1.209, 5.649), 0.015	2.423 (1.079, 5.443), 0.032
G3	3.154 (1.819, 5.469), <0.001	3.176 (1.808, 5.578), <0.001	3.289 (1.810, 5.975), <0.001
G4	2.319 (1.131, 4.758), 0.022	2.326 (1.124, 4.812), 0.023	2.646 (1.220, 5.742), 0.014
G5	5.882 (2.479, 13.959), <0.001	6.160 (2.441, 15.548), <0.001	5.767 (2.224, 14.952), <0.001
G6	9.448 (5.241, 17.031), <0.001	9.532 (5.220, 17.404), <0.001	10.258 (5.355, 19.653), <0.001
*p* for trend	<0.001	<0.001	<0.001
Male			
Total	1.642 (1.412, 1.908), <0.001	1.638 (1.404, 1.911), <0.001	1.714 (1.441, 2.039), <0.001
G1	Reference	Reference	Reference
G2	2.929 (1.043, 8.226), 0.041	3.586 (1.175, 10.950), 0.025	4.620 (1.408, 15.153), 0.012
G3	4.711 (2.118, 10.478), <0.001	5.562 (2.376, 13.020), <0.001	6.275 (2.490, 15.814), <0.001
G4	2.685 (0.969, 7.434), 0.057	2.870 (1.000, 8.234), 0.050	3.793 (1.191, 12.082), 0.024
G5	12.300 (3.900, 38.792), <0.001	13.188 (3.949, 44.040), <0.001	16.476 (4.531, 59.905), <0.001
G6	14.434 (6.259, 33.285), <0.001	15.570 (6.499, 37.304), <0.001	20.101 (7.481, 54.009), <0.001
*p* for trend	<0.001	<0.001	<0.001
Female			
Total	1.370 (1.171, 1.602), <0.001	1.386 (1.177, 1.631), <0.001	1.509 (1.247, 1.826), <0.001
G1	Reference	Reference	Reference
G2	2.214 (0.724, 6.770), 0.163	2.386 (0.751, 7.581), 0.141	1.385 (0.395, 4.848), 0.611
G3	2.131 (0.987, 4.602), 0.054	1.948 (0.885, 4.289), 0.098	2.133 (0.883, 5.152), 0.092
G4	2.067 (0.737, 5.795), 0.168	2.146 (0.751, 6.132), 0.154	3.130 (0.918, 10.667), 0.068
G5	1.550 (0.347, 6.916), 0.566	1.505 (0.288, 7.859), 0.628	1.022 (0.177, 5.916), 0.980
G6	6.071 (2.602, 14.165), <0.001	6.545 (2.691, 15.917), <0.001	10.266 (3.602, 29.259), <0.001
*p* for trend	0.003	0.004	0.001
*p*-Value for interaction^a^	<0.001	<0.001	<0.001

G1, normokalemia and bilateral normal on CT; G2, normokalemia and bilateral lesion on CT; G3, normokalemia and unilateral lesion on CT; G4, hypokalemia and bilateral normal on CT; G5, hypokalemia and bilateral lesion on CT; G6, hypokalemia and unilateral lesion on CT. Model 1 was adjusted for none. Model 2 was adjusted for age, sex (only for overall population), BMI, diabetes, eGFR, SBP, and DBP. Model 3 was adjusted for age, sex (only for overall population), BMI, diabetes, eGFR, SBP, DBP, TC, TG, HDL, LDL, FPG, HbA1c, and Hcy.

AVS, adrenal venous sampling; PA, primary aldosteronism; BMI, body mass index; eGFR, estimated glomerular filtration rate; SBP, systolic blood pressure; DBP, diastolic blood pressure; TC, serum total cholesterol; TG, triglyceride; HDL, high-density lipoprotein; LDL, low-density lipoprotein; FPG, fasting plasma glucose; HbA1c, glycosylated hemoglobin; Hcy, homocysteine.

a p-Value for interaction: two-way interaction of sex and the combination of CT findings and serum potassium on unilateral PA.

### Subgroup analysis

**Figure 1:**
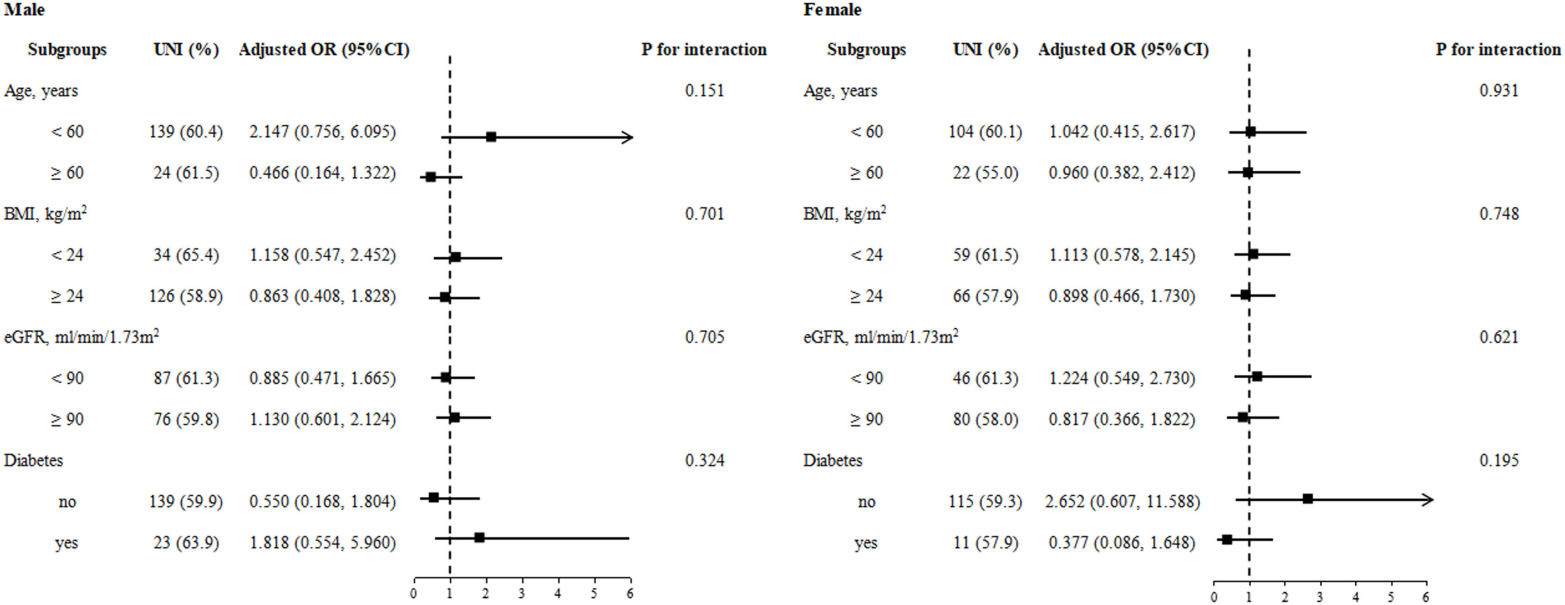
Stratified analyses by potential modifiers of the association between the combination of CT findings and serum potassium and unilateral PA by sex. Adjusted for age, BMI, diabetes, eGFR, SBP, DBP, TC, TG, HDL, LDL, FBG, HbA1c, and Hcy, if not be stratified.

In the stratified analyses, none of the variables, including age, BMI, eGFR, and diabetes, significantly modified the association between the prevalence of unilateral PA and the combination of CT findings and serum potassium among male and female participants (all *p* for interaction >0.05) (**Figure 1**).

## Discussion

Our study confirmed that the combination of CT findings and serum potassium has a great value for predicting the subtype of PA. The occurrence of unilateral lesions on CT and hypokalemia is significantly associated with an increased prevalence of unilateral PA. Moreover, we found that the association of subtype on AVS with the combination of CT findings and serum potassium was stronger in male participants, compared with female participants.

Primary aldosteronism is the most common but largely unrecognized cause of secondary hypertension (21). Unilateral adrenalectomy can normalize aldosterone excess, cure or improve hypertension, and prevent target organ damage in patients with unilateral PA, while bilateral disease is treated with MR antagonist (6, 7). Hence, distinguishing unilateral and bilateral PA is critical for selecting the most appropriate treatment. In the present study, the overall diagnostic concordance rate between CT findings and AVS outcomes was 51.5%, which is similar to previous studies (10–12). Although the current guidelines recommend using adrenal CT severing as the initial study for subtype testing, the specificity of CT findings could not be sufficient to allow surgical decision-making without AVS evidence. Indeed, we found that 38.0% (57/150) of patients with bilateral normal CT showed unilateral PA on AVS, while 22.4% (58/259) of patients with unilateral lesion on CT exhibited unilateral hyperaldosteronism in the contralateral adrenal gland. Research has revealed that thin-section high-resolution CT omits 27 cases of aldosterone-producing microadenoma among 69 cases of APA adrenal because CT cannot detect an adrenal mass less than 6 mm in diameter (22). Meanwhile, non-functioning adenoma is not unusual in PA patients (23, 24). It is indicated that AVS is necessary before surgery to avoid unnecessary or inappropriate surgery. The current guidelines recommend AVS as the gold standard test to lateralize the source of excessive aldosterone secretion. However, AVS is a high-cost, technically difficult, and invasive procedure and has a risk of complications such as adrenal vein rupture, hemorrhage, and thromboembolism (8). Considering the high prevalence of PA, subtype classification based on safe, inexpensive, and reliable methods is very important. Several models have been developed to predict PA subtypes based on clinical findings and with high specificity (13–19). The most widely accepted and classical model was the Küpers prediction score, which suggested that AVS could be omitted in patients with a typical Conn’s adenoma plus serum potassium <3.5 mmol/L or eGFR ≥ 100 ml/min/1.73 m^2^. In subjects with a score of at least 5, this criterion had a specificity of 100% (95% CI, 91%–100%) and a sensitivity of 53% (95% CI, 38%–68%) to predict a lateralized AVS (13). However, subsequent research showed the Küpers prediction score was not suitable for elderly and Chinese patients (19, 25). Since failure to correctly predict unilateral PA will result in inappropriate or unnecessary adrenalectomy, it seems more reasonable to develop a prediction model to identify patients with bilateral PA that could spare AVS. Kocjan et al. (14) first established a scoring system based on the combined criterion of serum potassium ≥3.5 mmol/L, post-SIT aldosterone <18 ng/dl, and either no or bilateral tumor found on CT imaging to predict bilateral PA, which saved an estimated 16% of the patients from unnecessary AVS. Xiao et al. (18) developed and validated a nomogram that was able to predict the probability of IHA based on BMI, serum potassium, and CT findings, and the specificity reached 100% when the threshold increased to a probability of 90%. Although the above studies incorporated different clinical indicators, CT findings and serum potassium served as shared predictors for subtype diagnosis of PA patients. Recent research showed that the prevalence of unilateral PA on AVS in patients with unilateral disease on CT with hypokalemia, unilateral disease on CT with normokalemia, bilateral normal on CT with hypokalemia, and bilateral normal on CT with normokalemia was 70.6%, 38.1%, 23.8%, and 6.2%, respectively (12). Our study further demonstrates the value of this prediction score for identifying patients who should undergo AVS before surgery by showing that patients with unilateral lesions on CT and hypokalemia had a high likelihood of lateralization on AVS.

Notably, we found that sex can modify the relationship between the subtype of PA and the combination of CT findings and serum potassium. The presence of abnormality on CT and hypokalemia was significantly associated with an increased incidence of unilateral PA among male participants, but not stably in female participants. Previous studies have shown the relationship between sex and the subtype distribution of PA, but the conclusions of these studies have been controversial. A number of studies reported that women had a higher incidence of bilateral PA (14, 16, 17, 26). In contrast, the literature indicated that the proportion of male participants was significantly higher in patients with IHA compared with those with APA (18). However, possible mechanisms for the positive or negative relationship were not mentioned in the literature. Meanwhile, several previous studies did not observe a difference between men and women in the subtype distribution in AVS (13, 15, 27), which is consistent with our current finding. These conflicting results might be attributed to the different definitions of PA subtype and interpretation of AVS in the above studies and the wide variation of study populations in different regions.

There is limited information available about how sex influences serum potassium and adrenal CT results in PA patients. Consistent with the present study, Akasaka et al. (26) found that the incidence of hypokalemia was higher in men. Although there was no difference between men and women with unilateral PA in PAC, we found that male patients with unilateral PA had lower serum potassium levels than female patients. In order to accurately predict the probability of APA in patients with unilateral adrenal nodules, He et al. (28) included men as a positive indicator for a nomogram. Taken together, compared with female patients, male patients with unilateral PA have more obvious clinical features; therefore, the positive correlation between unilateral PA and the combination of CT findings and serum potassium is more significant in men. Research based on the Japan Primary Aldosteronism Study indicated that sex may have a clinically significant impact on the distribution of subtypes differently among the generations, even if it is not a definitive factor in determining the age at the onset of PA for each subtype (26). Moreover, a study demonstrated that women had a higher prevalence of KCNJ5 mutations, which are present in 39.3% of APA patients (29). Furthermore, female sex is associated with poorer renal outcomes in patients receiving spironolactone for bilateral PA (30). Although the possible mechanism is unclear, sex differences should be considered when applying CT findings combined with serum potassium to predict subtypes of PA.

### Limitations

There are several limitations to this study. The study was conducted only on a specific population. Our results might not be generalizable to other patient cohorts. In addition, the retrospective nature of the study limited its usefulness. Although our study included more participants compared to most previous studies, prospective studies are still needed to confirm and validate the current results. The PA subtype was based on the outcomes of bilaterally simultaneous catheterization AVS without ACTH stimulation in this study. The criterion used for unilateral hyperaldosteronism was LI > 2. Although this criterion is the most commonly used threshold recommended by current guidelines, the lateralization of AVS is not completely consistent with the subtype of PA. Thus, the subtype diagnosis of PA should be revised by the results of postoperative histological examination and treatment outcomes.

## Conclusion

In conclusion, we found that the association of subtype on AVS with the combination of CT findings and serum potassium was stronger in male participants, compared with female participants.

## Data availability statement

The raw data supporting the conclusions of this article will be made available by the authors, without undue reservation.

## Ethics statement

The studies involving humans were approved by The Medical Research Ethics Committee of The Second Affiliate Hospital of Nanchang University. The studies were conducted in accordance with the local legislation and institutional requirements. The participants provided their written informed consent to participate in this study.

## Author contributions

YW: Conceptualization, Writing – original draft, Writing – review & editing. ZW: Data curation, Methodology, Writing – review & editing. JR: Data curation, Methodology, Writing – review & editing. HH: Data curation, Methodology, Writing – review & editing. ZC: Data curation, Methodology, Writing – review & editing. CH: Data curation, Methodology, Writing – review & editing. QP: Data curation, Methodology, Writing – review & editing. PL: Conceptualization, Funding acquisition, Writing – original draft, Writing – review & editing.
